# Emotion and the Cardiovascular System: Postulated Role of Inputs From the Medial Prefrontal Cortex to the Dorsolateral Periaqueductal Gray

**DOI:** 10.3389/fnins.2018.00343

**Published:** 2018-05-24

**Authors:** Roger Dampney

**Affiliations:** School of Medical Sciences (Physiology) and Bosch Institute, University of Sydney, Sydney, NSW, Australia

**Keywords:** periaqueductal gray, medial prefrontal cortex, Brodmann cortical area 10, cardiovascular regulation, respiratory activity, defensive behavior, cognition

## Abstract

The midbrain periaqueductal gray (PAG) plays a major role in generating different types of behavioral responses to emotional stressors. This review focuses on the role of the dorsolateral (dl) portion of the PAG, which on the basis of anatomical and functional studies, appears to have a unique and distinctive role in generating behavioral, cardiovascular and respiratory responses to real and perceived emotional stressors. In particular, the dlPAG, but not other parts of the PAG, receives direct inputs from the primary auditory cortex and from the secondary visual cortex. In addition, there are strong direct inputs to the dlPAG, but not other parts of the PAG, from regions within the medial prefrontal cortex that in primates correspond to cortical areas 10 m, 25 and 32. I first summarise the evidence that the inputs to the dlPAG arising from visual, auditory and olfactory signals trigger defensive behavioral responses supported by appropriate cardiovascular and respiratory effects, when such signals indicate the presence of a real external threat, such as the presence of a predator. I then consider the functional roles of the direct inputs from the medial prefrontal cortex, and propose the hypothesis that these inputs are activated by perceived threats, that are generated as a consequence of complex cognitive processes. I further propose that the inputs from areas 10 m, 25 and 32 are activated under different circumstances. The input from cortical area 10 m is of special interest, because this cortical area exists only in primates and is much larger in the brain of humans than in all other primates.

## Introduction

An animal's survival depends upon being able to respond appropriately to stimuli that may signal a threat, such as the presence of a predator. In the course of evolution, several different defense systems have evolved. One of the most phylogenetically old systems is subserved by neural pathways within the basal ganglia and midbrain colliculi (homologous to the optic tectum in fish, amphibian, reptiles and birds). This system, which is not dependent on inputs from the cortex and thus appears to be subconscious, produces highly coordinated and stereotyped behavioral responses to visual, auditory and somatosensory inputs, accompanied by appropriate cardiovascular and respiratory effects (Dean et al., 1989; Dampney, 2015, 2016; Müller-Ribeiro et al., 2016). The particular type of stereotyped response that is evoked (e.g., orienting, pursuit or escape) depends upon the precise pattern of inputs that trigger the response. This type of defense response is advantageous in a situation where immediate action is required. On the other hand, such responses lack the flexibility that would be essential in a situation where the threat is sustained and changing—in that case, cognitive appraisal of the threat is required to ensure that the response is most appropriate to the particular situation.

Apart from the basal ganglia and colliculi, many other brain regions also play important roles in generating responses to threatening stimuli in mammals. These regions include the medial prefrontal cortex, amygdala, various hypothalamic nuclei, and the midbrain periaqueductal gray (PAG) (Dampney, 2015). The specific roles of these different nuclei in generating behavioral and physiological responses to threatening stimuli have been discussed in a number of recent reviews (Dampney et al., 2013; Fontes et al., 2014; Carr, 2015; Dampney, 2015; LeDoux and Pine, 2016; Myers, 2017). This review, however, shall focus on the role of the dorsolateral (dl) portion of the PAG, which, as I shall explain in the following sections, appears to be a critical component of the central pathways subserving responses to inputs arising from the medial prefrontal cortex.

## Anatomical and functional properties of the dlPAG

The dlPAG is one of four longitudinal columns within the PAG, the others being the dorsomedial (dmPAG), lateral (lPAG), and ventrolateral (vlPAG) columns. These different components of the PAG differ greatly with respect to their functional properties, anatomical connections and chemical properties (Carrive, 1993; Bandler and Shipley, 1994; Bandler et al., 2000; Keay and Bandler, 2001; Vianna and Brandão, 2003; Dampney et al., 2013). Activation of neurons in the lPAG and dlPAG generate active defensive behavioral responses, which are characterized by increased somatomotor activity, and cardiovascular and respiratory changes that have the effect of increasing the blood flow and supply of oxygen to active skeletal muscles. Such a response can be triggered by an escapable stimulus, such as the presence of a predator or cutaneous pain (Bandler et al., 2000; Keay and Bandler, 2001). In contrast, the vlPAG is critical for the expression of passive behavioral responses, which are characterized by decreased somatomotor activity, accompanied by decreased arterial pressure and heart rate. Such responses are believed to be triggered by inescapable stimuli, such as hemorrhage or visceral pain (Bandler et al., 2000; Keay and Bandler, 2001).

**Figure 1:**
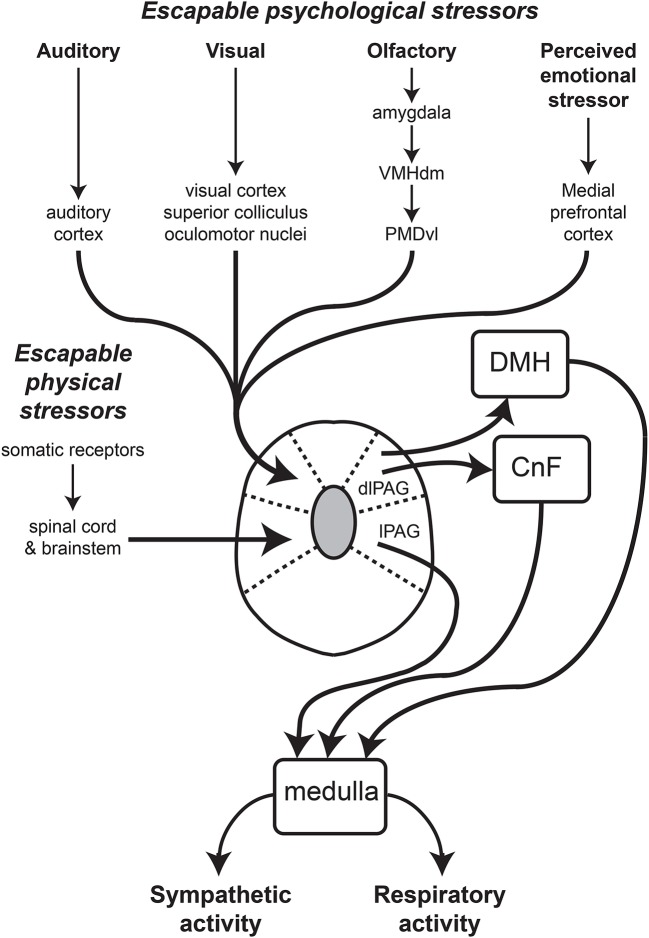
Schematic diagram showing major inputs to the dlPAG and the proposed output pathways subserving the co-ordinated changes in sympathetic vasomotor and respiratory activity regulated by the dlPAG. The lines with arrows indicate connections, that could be either direct (monosynaptic) or indirect (polysynaptic). Other abbreviations: CnF, cuneiform nucleus; DMH, dorsomedial hypothalamus; lPAG, lateral part of the periaqueductal gray. Modified from Dampney et al. (2013).

The functional differences between the different subdivisions of the PAG are also reflected by their anatomical connections (Figure 1). In particular, the dlPAG is distinctly different from the other PAG subdivisions with respect to both its afferent inputs and efferent outputs (Dampney et al., 2013). Studies in the rat have shown that the dlPAG, but not the other PAG subdivisions, receives direct inputs from the primary auditory cortex and from the secondary visual cortex (Benzinger and Massopust, 1983; Newman et al., 1989) as well as from the superior colliculus (Rhoades et al., 1989), which in turn receives both visual and auditory inputs (May, 2006). There are also inputs to the dlPAG but not other PAG subdivisions from the nucleus praepositus hypoglossi and the periparabigeminal nucleus in the lower brainstem (Klop et al., 2005, 2006). Both of these nuclei have a role in the control of eye movements, and it has been suggested that inputs from these nuclei allow neurons in the PAG to distinguish between visual signals generated by objects that have moved into the visual field from visual signals caused only by eye movements (Klop et al., 2006).

A very dense input to the dlPAG, but not other PAG subdivisions arises from the dorsal premamillary nucleus (PMD) in the hypothalamus, especially its ventrolateral portion (Motta et al., 2009). The ventrolateral PMD receives inputs from neurons within the medial and basomedial nuclei in the amygdala, relayed via the hypothalamic ventromedial nucleus (Motta et al., 2009). The projections from these amygdaloid nuclei to the dlPAG are believed to convey signals relating to the presence of a predator (Motta et al., 2009). Consistent with this, blockade of neurons in the PMD reduces the behavioral defensive response to a predator or predator odor alone (Markham et al., 2004; Blanchard et al., 2005; Motta et al., 2009).

There are no direct inputs to the dlPAG from any part of the spinal cord, whereas all other PAG subdivisions receive direct inputs from cervical, thoracic and lumbar segments of the spinal cord (Keay and Bandler, 2001; Dampney et al., 2013). Similarly, the lPAG and vlPAG, but not the dlPAG, receive inputs from the spinal trigeminal nucleus and nucleus of the solitary tract (NTS), respectively (Keay and Bandler, 2001; Dampney et al., 2013). In summary, the dlPAG, but not other parts of the PAG, receives direct inputs that convey visual, auditory and olfactory signals, but do not receive direct inputs conveying signals from visceral or somatic receptors. In contrast, the lPAG and vlPAG both receive major inputs from visceral and somatic receptors, but not direct inputs from brain regions that receive visual, auditory or olfactory inputs.

Consistent with the fact that the dlPAG receives visual, auditory and olfactory signals, exposure to a cat generates strong c-fos expression in the dlPAG, but not in other PAG subdivisions (Canteras and Goto, 1999). Furthermore, in conscious rats the defensive behavioral responses of freezing or flight is evoked with very low intensity stimulation of the dlPAG, whereas such stimulation is ineffective in other parts of the PAG (Bittencourt et al., 2004). Finally, in anesthetized rats, stimulation of the dlPAG evokes increases in sympathetic activity and respiration (Iigaya et al., 2010). Thus, the available anatomical and functional evidence indicates that the dlPAG is a major component of the central pathways that generate behavioral defensive responses to an external perceived threat, supported by appropriate autonomic and respiratory effects.

In contrast to all other PAG regions, the dlPAG is notable for the absence of labeled neurons following injections of retrogradely transported tracers into the medulla or spinal cord (Van Bockstaele et al., 1991; Cowie and Holstege, 1992; Dampney et al., 2013; Figure 1). Instead, neurons in the dlPAG influence autonomic and respiratory activity via direct and indirect projections to the dorsomedial hypothalamus (Horiuchi et al., 2009; Dampney et al., 2013; Dampney, 2015).

In humans, Ezra et al. (2015) used magnetic resonance imaging and probabilistic tractography to determine the functional connectivity between different PAG subregions and other brain regions. These authors found that there was functional connectivity between the dlPAG and two lower brainstem regions, the parabrachial/Kölliker-Fuse complex and the ventrolateral medulla. As Ezra et al. (2015) point out, however, the demonstrated functional connectivity may be direct or indirect. In fact, Ezra et al. (2015) also found that there was functional connectivity between the dlPAG and the midbrain cuneiform nucleus, which in rats has been shown to project to the ventrolateral medulla (Verberne, 1995). In addition, a direct projection from the dlPAG to the parabrachial/Kölliker-Fuse complex has also been demonstrated in rats (Krout et al., 1998). Thus, the connections of the dlPAG with brainstem regions as identified in humans by Ezra et al. (2015) is entirely consistent with the anatomical connections of the dlPAG as demonstrated in rats.

## Functions of projections from the medial prefrontal cortex to the dlPAG

**Figure 2:**
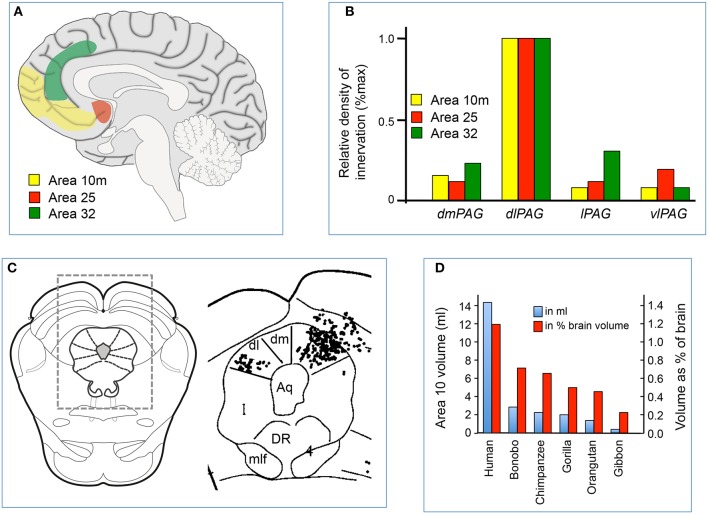
**(A)** Sagittal midline section of the human brain showing the location of cortical areas 10 m, 25 and 32. **(B)** Relative density of labeled axonal terminals in the dorsomedial (dm), dorsolateral (dl), lateral (l), and ventrolateral (vl) parts of the PAG, following injection of anterograde tracer into areas 10 m, 25 and 32 in the medial prefrontal cortex of the macaque. Modified from An et al. (1998), with permission; **(C)** Example of distribution of labeled terminals in the PAG and surrounding areas following injection of anterograde tracer into areas 10 m in one experiment. The location of the area on the right is indicated by the rectangle over a standard section of the midbrain on the left. Modified from An et al. (1998). **(D)** Relative volumes of area 10 m in different primate species, expressed both as an absolute measure (in ml) or relative measure (% of brain volume). Modified from Semendeferi et al. (2001), with permission.

There are projections from the medial prefrontal cortex to all PAG subregions (An et al., 1998; Floyd et al., 2000), but in the rat projections from the anterior cingulate and caudal prelimbic cortices terminate predominantly in the dlPAG (Floyd et al., 2000; Gabbott et al., 2005). Similarly, it has been shown in the macaque monkey that there are strong direct projections from the medial prefrontal cortex to the dlPAG, specifically from Brodmann area 10 (particularly its medial portion, area 10 m), area 25 and area 32 (An et al., 1998; Freedman et al., 2000; Figures 2B,C). In comparison, projections from these regions to other PAG subregions are of much smaller magnitude (Figures 2B,C). In the primate, Brodmann areas 25 and 32 are homologous to the infralimbic and caudal prelimbic cortices in the rat, but area 10 m has no apparent homologue in the rat or in other non-primate species (Floyd et al., 2000).

There have been few studies of the functions of the direct projections from the medial prefrontal cortex to the dlPAG. A recent study in mice, however, used optogenetics to selectively manipulate the projection from the medial prefrontal cortex to the dorsal PAG (Franklin et al., 2017). This study found that selective inhibition of this pathway produced a behavioral response that mimicked social defeat. In this study, the dorsal PAG referred to the entire dorsal half of the PAG, which includes the dmPAG, dlPAG, and lPAG, and so it is not possible on the basis of this study to determine whether the observed behavioral effects were due to activation of the pathway from the medial prefrontal cortex to the dlPAG, rather than the dmPAG or lPAG. Indeed, an earlier study by Faturi et al. (2014) found, on the basis of studies using c-fos expression and pharmacological manipulations that the dmPAG was an important node for the expression of contextual responses to social defeat.

### Area 25 (subgenual cingulate cortex)

There is considerable evidence from studies in humans that area 25 (also called the subgenual cingulate cortex) has an important role in regulating emotions and the physiological response to negative emotions and threatening stimuli (Phelps et al., 2004; Milad et al., 2007). In particular, area 25 is involved in the extinction of a behavioral fear response to a conditioned stimulus, which occurs when a conditioned stimulus is presented repeatedly, without the unconditioned stimulus (Phelps et al., 2004; Milad et al., 2007). It is also interesting to note that area 25 is metabolically overactive in patients with treatment-resistant depression (Mayberg et al., 2000).

There are few studies in primates of the role of area 25 in cardiovascular regulation. In the rat, however, it has been shown that direct stimulation of the homologous region (infralimbic cortex) attenuates the cardiovascular response (increase in blood pressure and heart rate) evoked by air-jet stress (Müller-Ribeiro et al., 2012). Stimulation of the infralimbic cortex does not alter cardiovascular variables under baseline conditions (Müller-Ribeiro et al., 2012). These observations are compatible with the possibility that the direct projection from the infralimbic cortex (or area 25 in primates) inhibits neurons in the dlPAG that normally generate cardiovascular responses to threatening stimuli. Inhibition of the infralimbic cortex has no effect on these responses, however, suggesting that the neurons in the infralimbic cortex that can inhibit stress-evoked responses are not tonically active under resting conditions (Müller-Ribeiro et al., 2012).

### Area 32 (dorsal anterior cingulate cortex)

In contrast to area 25, in humans activation of area 32 (also called the dorsal anterior cingulate cortex) is correlated with the magnitude of conditioned fear responses (Milad et al., 2007). Consistent with this, in rats stimulation of the homologous region (prelimbic cortex) increases conditioned fear responses (Vidal-Gonzalez et al., 2006) whereas inactivation of the prelimbic cortex reduces these responses (Corcoran and Quirk, 2007).

In a neuroimaging study in humans, Critchley et al. (2000) found that activity in area 32 correlated with increases in blood pressure during periods of psychological stress (mental arithmetic). This observation led to the prediction that lesions of area 32 would impair cardiovascular responses to psychological stress, which was tested in a group of three patients with brain lesions that included area 32 (Critchley et al., 2003). All three patients had average or above-average cognitive ability as measured in a range of tests, but all three showed reduced cardiovascular responses to psychological stress (mental arithmetic). Thus it appears that area 32 generates physiological responses associated with cognitive demands, but is not involved in the cognitive process itself.

Apart from cognition, area 32 is also believed to be a region that generates responses to pain (Etkin et al., 2011; Shackman et al., 2011; Jahn et al., 2016). There are two general theories concerning the role of area 32 in pain processing. One theory, the neural alarm hypothesis, proposes that area 32 generates immediate responses to painful stimuli, without cognitive appraisal of this stimulus (Lieberman and Eisenberger, 2015). The second theory, called the adaptive control hypothesis, proposes that area 32 acts as a hub that processes aversive stimuli regardless of modality (e.g., pain, or negative affect) and then generates an appropriate behavioral response (Shackman et al., 2011). The adaptive control hypothesis implies that cognitive processing is required to generate an appropriate response (Jahn et al., 2016). The results of recent studies using brain imaging support the neural alarm hypothesis, in that they show that pain activates a region within area 32 (Lieberman and Eisenberger, 2015; Jahn et al., 2016) that is separate from the region activated by cognitive processing. It is conceivable that neurons within area 32 that are activated by pain also project to the dlPAG, and trigger behavioral and cardiovascular responses via this pathway, but no studies have been performed to test that possibility.

### Area 10

Area 10 is located in the most rostral part of the prefrontal cortex (Figure 2A), and is sometimes referred to as the frontopolar cortex or the rostral prefrontal cortex. In recent years area 10 has attracted much interest, for several reasons. First, as mentioned above, it is a brain structure that either does not exist in non-primate species, or is so rudimentary that it cannot be identified. Secondly, the volume of area 10 (either as an absolute measure, or as a percentage of the volume of the whole brain) is much greater in humans than in other primates (Semendeferi et al., 2001; Figure 2D). Thirdly, virtually all the afferent inputs to area 10 originate from higher-order association areas, suggesting this region has an important role in cognition (Barbas and Pandya, 1989; Burman et al., 2011). Finally, the maturation of area 10 occurs late in the postnatal period (Dumontheil, 2014), suggesting that it is a region where experience may affect its development, consistent with a role in higher-order cognition.

Less is known about the specific functions of area 10 than other parts of the medial prefrontal cortex. Nevertheless, some information is available. Monkeys in which area 10 was lesioned had a deficit in their ability to explore alternative goals when distracted during an ongoing behavioral task (Boschin et al., 2015; Mansouri et al., 2015). Such studies have led to the hypothesis that neurons in area 10 have an exploratory role, i.e., monitor the environment for indications that it may be advantageous to abandon the current ongoing task and pursue a different task (Mansouri et al., 2017).

Brain imaging studies in human subjects have shown that area 10 is activated when subjects perform tasks that require them to recall specific events from the past (Lepage et al., 2000). Furthermore, the medial part of area 10 (area 10 m) is specifically activated when subjects are presented with an emotionally charged dilemma that require them to make a choice between two different courses of action, each of which may affect the welfare of others (Greene et al., 2001). On the basis of these observations, Allman et al. (2002) have proposed that in humans area 10 compares the current behavioral state with past experiences, and then makes a choice with regard to future behavior. Taken together, studies in monkeys and humans suggest that area 10 continually compares the ongoing behavioral state with alternative behaviors, to determine if alternative behaviors may be more advantageous in the light of changes in the external environment together with past experiences. The fact that area 10 m (which has a strong direct projection to the dlPAG) (Figures 2B,C) is specifically activated under conditions where such comparisons generate an emotional response is particularly interesting, given that emotional responses are associated with autonomic and respiratory effects.

## Conclusions and future directions

Cortical areas 25, 32, and 10 m in the primate medial prefrontal cortex are all involved in the regulation of behavioral responses to threatening or emotional stimuli, either real or perceived. Although there are differences in their precise functions, a common property of these regions is that all three have major direct projections to the dlPAG, but not to other PAG regions. As discussed previously, there is strong evidence that the dlPAG plays an important role in regulating behavioral responses to psychological stressors, accompanied by appropriate autonomic and respiratory changes (Dampney et al., 2013). Thus, the anatomical and functional evidence taken together suggests that the inputs to the dlPAG from areas 25, 32, and 10 m in the medial prefrontal cortex are major components of the central networks coupling emotional stimuli to behavioral and physiological responses, as previously suggested (Bandler et al., 2000; Keay and Bandler, 2001).

As discussed above, apart from inputs from the medial prefrontal cortex, the dlPAG also receives inputs related to auditory, visual and olfactory signals (Figure 1) that indicate a direct external threat such as the presence of a predator. In summary, the available evidence indicates that the dlPAG is a site of convergence of inputs arising from sensory receptors indicating a direct external threat, as well as inputs from the medial prefrontal cortex indicating a perceived threat based on more complex cognitive processing.

There are many unresolved issues concerning the relationship between cortical areas 25, 32, and 10 m and the dlPAG. First, while there is good evidence that areas 25 and 32 can modulate behavioral and cardiovascular responses to emotional stimuli, there is no direct evidence that these effects are mediated by direct projections from these areas to the dlPAG. In the case of neurons in area 10 m that project directly to the dlPAG, there is no information available regarding their potential role in cardiovasculat regulation. Secondly, even if the projections from areas 25, 32, and 10 m to the dlPAG do mediate behavioral and physiological responses to emotional stimuli, are these projections the only route by which such responses are generated? Finally, are the neurons within the dlPAG functionally homogeneous, or are their subsets of dlPAG neurons that receive differential inputs from areas 25, 32, and 10 m in the medial prefrontal cortex?

These questions may be answered, at least in part, by future studies using optogenetics. For example, a number of recent studies in rodents have examined the effects of selective activation or inhibition of projections from the medial prefrontal cortex to a specific target (e.g., the dorsal raphe nucleus, Warden et al., 2012), or projections to specific subregions of the PAG (e.g., to the ventral PAG from the medial preoptic area, Park et al., 2018 or the lPAG from the lateral hypothalamus, Li et al., 2018). The same methodology could be applied to selectively activate the projection to the dlPAG in rodents from the infralimbic cortex (which is analogous to cortical area 25 in primates) or from the dorsal ACC (which is analogous to cortical area 25 in primates). Such experiments would reveal the behavioral and physiological effects of activation or inhibition of these pathways in rodents. In regard to human studies, recent improvements in resolution of brain imaging technology together with new methods for determining functional connectivity in the brain have the potential for identifying the function of specific connections between the medial prefrontal cortex and subregions of the PAG, including the dlPAG (Linnman et al., 2012).

Although much remains unknown, the evidence so far suggests that the connections from the medial prefrontal cortex to the dlPAG have a critical role in generating behavioral, cardiovascular and other physiological responses associated with emotional stimuli that require cognitive appraisal.

## Author contribution

The author confirms being the sole contributor of this work and approved it for publication.

### Conflict of interest statement

The author declares that the research was conducted in the absence of any commercial or financial relationships that could be construed as a potential conflict of interest.
